# Reduction en masse of Inguinal Hernia in a 2-Month-Old Male Infant

**DOI:** 10.1055/a-2280-9708

**Published:** 2024-03-26

**Authors:** Masato Kojima, Ryo Touge, Sho Kurihara, Isamu Saeki, Shinya Takahashi

**Affiliations:** 1 Department of Pediatric Surgery, Hiroshima University Hospital, Hiroshima, Japan; 2 Department of Biomedical Science, Natural Science Center for Basic Research and Development, Hiroshima University, Hiroshima, Japan; 3 Department of Surgery, Graduate School of Biomedical and Health Sciences, School of Medicine, Hiroshima University, Hiroshima, Japan

**Keywords:** reduction en masse, inguinal hernia, pediatrics, infant

## Abstract

Reduction en masse is the reduction of the hernial sac into the preperitoneal space, with a loop of bowel remaining trapped at the neck of the hernial sac. This complication is rare, usually associated with inguinal hernias, and is characterized by the absence of a noticeable bulge in the groin. The patient was a 2-month-old male infant and presented with a nonreducible bulge in his left groin, and incarceration of the left inguinal hernia was diagnosed. Manual reduction was performed, and the hernia bulge became less noticeable. He was admitted, and laparoscopic percutaneous extraperitoneal closure was scheduled for the next day. The laparoscopy revealed remarkably dilated intestines, serous ascites, and an ischemic intestine in the left groin. A laparotomy was performed and revealed reduction en masse of the left inguinal hernia with a strangulated ileum at its neck. We made an incision at the neck, followed by the resection of 20-cm long strangulated ileum. The patient's condition was unstable on the day of operation, but the postoperative period was uneventful, and the left inguinal hernia was repaired, 11 months after the operation. Reduction en masse in pediatrics is significantly rare but when it occurs, the diagnosis can be delayed and occasionally the patient will be life-threatening. To avoid reduction en masse, it is crucial to perform the reduction gently and confirm the absence of a hernia sac in the preperitoneal space containing a loop of bowel by ultrasound scanning. Moreover, contrary to common practice, overnight observation and close monitoring will avoid missing a late presentation, leading to timely interventions.

## Introduction

Inguinal hernia is one of the most common conditions encountered by pediatricians and surgeons. Emergency cases often result from incarceration or intestinal obstruction. Delaying treatment can lead to strangulation and ischemia of the intestine, sometimes accompanied by peritonitis. 1 Typically, incarcerated inguinal hernias without signs of strangulation are managed by manual reduction, which is generally effective and rarely leads to complications.

Reduction en masse is the reduction of the hernial sac into the preperitoneal space, with a loop of bowel remaining trapped at the neck of the hernial sac. 2 This complication is rare following hernia reduction, usually associated with inguinal hernias, and is characterized by the absence of a noticeable bulge in the groin. 3 The treating physician believes that successful reduction has been achieved and discharges the patient. However, without prompt intervention, strangulation and intestinal ischemia could develop.

We present a life-threatening case involving an infant with reduction en masse, necessitating resection of the strangulated intestine. This represents the youngest reported case of reduction en masse to date.

## Case Report

**Fig. 1:**
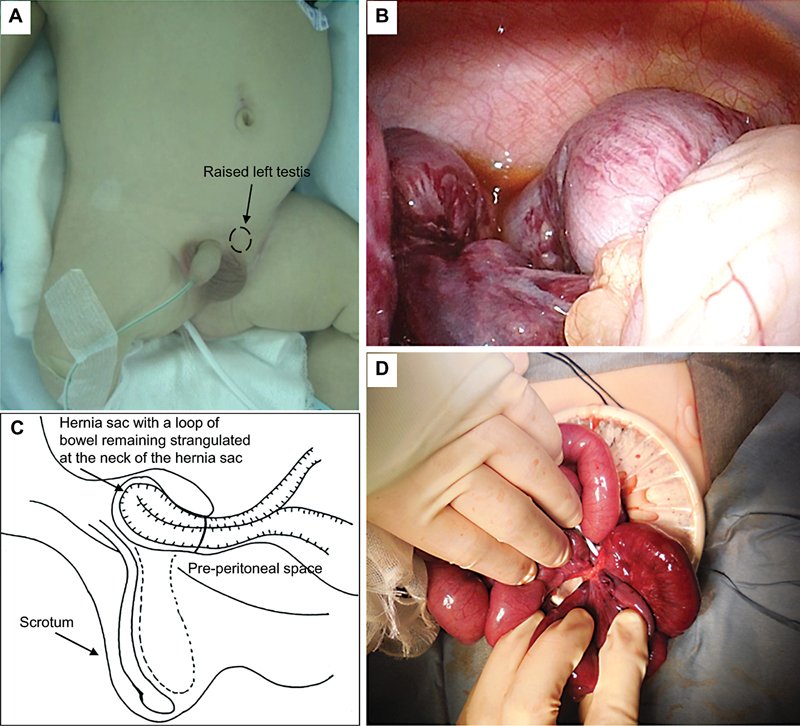
( **A** ) After manual reduction of the hernia, the hernia bulge was not appreciable, but the left testis was raised to the proximal of the inguinal canal (arrow). ( **B** ) Laparoscopy revealed a remarkably dilated intestine, serous ascites, and ischemic intestine at the left groin, which could not be retrieved to the surgical field. ( **C, D** ) A lower midline laparotomy was performed, and the hernia sac with a loop of bowel remaining strangulated at the neck of the hernia sac in preperitoneal space was noted. Incision of the thickened neck of the hernia sac, resection of the strangulated ileum, approximately 2 cm before the ileocecal valve, and end-to-end anastomosis were performed.

The patient, a 2-month-old male infant, was born at 37 weeks of gestation with birth weight of 2.5 kg and suffered from duodenal atresia. An open diamond-shaped duodenoduodenostomy was performed on the second day of life. Additionally, the patient had a left inguinal hernia and right undescended testis, with surgical intervention planned at the age of 12 months. The patient presented with a nonreducible bulge in his left groin on the night of the day before admission and showed irritability. The bulge gradually became hard and swollen, and he cried when the bulge was touched. Ultrasonography showed incarceration of intestine in the left inguinal hernia. Although manual reduction was challenging, the hernia bulge became less noticeable without pain, medication, and sedation. However, the left testis was positioned higher in the inguinal canal ( Fig. 1 ). He was admitted, and we scheduled laparoscopic percutaneous extraperitoneal closure for the next day. While hospitalized, the patient experienced abdominal distention and several times of nonbilious vomiting after breastfeeding. The laparoscopy was done the next day, and it revealed remarkably dilated intestines, serous ascites, and an ischemic intestine in the left groin ( Fig. 1 ). During the operation, the patient was head down positioned and we tried to retrieve the ischemic intestine by pulling this by grasp forceps from inside and pushing the bulge from outside but the ischemic intestine could not be retrieved ( Fig. 1 ). A lower midline laparotomy was performed, revealing reduction en masse of the left inguinal hernia with a strangulated ileum at its neck ( Fig. 1 ). We made an incision at the neck of the hernia sac, followed by the resection of the strangulated ileum, approximately 2 cm proximal to the ileocecal valve, and performed an end-to-end anastomosis. The internal inguinal ring was secured with sutures from the abdominal side, and the retractile left testis was fixed to the scrotum. The postoperative period was uneventful, and the patient was discharged on postoperative day 15. However, during follow-up, a recurrence of the left inguinal hernia was observed. We considered laparoscopic percutaneous extraperitoneal closure might be technically difficult because of adhesion around the internal inguinal ring. Therefore, the hernia was repaired using the Mitchell Banks procedure, the hernia sac was isolated through the external inguinal ring and ligated at the level of preperitoneal fat without opening inguinal canal, 4 and orchiopexy for the right testis was also performed at the age of 13 months, 11 months after the operation for reduction en masse.

## Discussion

Reduction en masse was first reported by Luke in 1843, 5 with an incidence of 1 in 13,000 hernias. 6 Reduction en masse is likely to occur in conditions with a mobile hernia sac in the inguinal canal, a narrow and fibrous neck of the hernia sac caused by repeated manual reduction over time, and a mobile parietal peritoneum surrounding the deep hernia ring, creating space for the hernia sac to become displaced while a loop of bowel remains incarcerated. 3 In pediatrics, inguinal hernia rarely presents with a long-standing history, and the hernia sac tends to adhere tightly to adjacent structures, such as the ductus deferens and testicular vessels. 6 In fact, the incidence of reduction en masse in pediatric patients may be significantly lower than 1 in 13,000 adult hernias, and only three pediatric cases of reduction en masse have been previously reported ( Table 1 ). Moreover, when reduction en masse occurs, a painful mass can sometimes be felt in the proximal inguinal canal or above the inguinal ring, and older children could complain of the symptom, but in younger children, especially in infants, as in our case, the symptom can be ambiguous and diagnosis of reduction en masse could be difficult. 7 In our case, the patient showed irritability, but the hernia bulge became less noticeable, and we misdiagnosed as hernia reduction had been successfully completed. The incarceration was not released, and strangulation and peritonitis proceeded. It is important to be aware of the disease for early diagnosis and prevention of such complications. In our case, the mobility of the parietal peritoneum surrounding the deep hernia ring and forceful reduction of the incarcerated hernia might have facilitated the displacement of the hernia sac into the preperitoneal cavity. The elevation of the left testis proximal to the inguinal canal following manual hernia reduction could be indicative of reduction en masse in pediatric patients, where adjacent structures adhere to the hernia sac and move with it. To ensure early detection and prevention of reduction en masse, ultrasound scanning should be employed to rule out its presence. However, in the report from the adult cases, ultrasound scanning offers high diagnostic accuracy for reduction en masse, with 86% sensitivity and 77% specificity. 7

**Table 1:** Reported cases of reduction en masse in pediatrics

Case	Author	Year	Age/sex	Type of hernia	Duration of hernia	Symptom	Day from reduction to operation (d)	Enterectomy	Procedure for hernia repair	The expected cause of reduction en masse
1	Olguner et al 3	2000	13 y/M	Inguinal hernia (right)	15 d	Abdominal pain, vomiting, painful bulge at inguinal area	< 1	Not done	High ligation via inguinal incision	Lack of adherence of hernia sac to the neighboring structure (mobile hernia sac)
2	Bernie et al 2	2012	7 y/M	Inguinal hernia (right)	N/A	Abdominal pain, vomiting, painful bulge at inguinal area	< 1	Not done	High ligation via inguinal incision	Lack of adherence of hernia sac to the neighboring structure (mobile hernia sac)
3	Yano et al 6	2022	10 mo/F	Inguinal hernia (left)	High ligation via inguinal incision of bilateral inguinal hernia at 4 mo old	Vomiting, bulge at inguinal area	1	Not done	Laparoscopic percutaneous extraperitoneal closure and iliopubic tract repair	Lack of adherence of hernia sac to the neighboring structure by previous Potts procedure (mobile hernia sac)
4	Our case		2 mo/M	Inguinal hernia (left)	2 mo	Vomiting, bulge at inguinal area	1	Done	High ligation via inguinal incision 11 mo after the operation for reduction en masse	Mobile parietal peritoneum surrounding deep hernia ring and forceful reduction of incarcerated hernia

Abbreviation: N/A, not available.

## Conclusion

When faced with the challenge of manually reducing an inguinal hernia, it is crucial to perform the reduction gently. To ensure early detection and prevention of reduction en masse, the absence of a hernia sac in the preperitoneal space containing a loop of bowel should be confirmed by ultrasound. Overnight observation and close monitoring may avoid a late presentation and timely interventions, even without an appreciable bulge in the groin.
